# Biosynthesis of a Novel Antibacterial Dipeptide, Using Proteases From South American Native Fruits, Useful as a Food Preservative

**DOI:** 10.3389/fnut.2021.685330

**Published:** 2021-06-28

**Authors:** Mauricio Adaro, Grisel Bersi, Juan Manuel Talia, Claudia Bernal, Fanny Guzmán, Diego Vallés, Sonia Barberis

**Affiliations:** ^1^Laboratorio Control de Calidad y Desarrollo de Bromatología, Facultad de Química, Bioquímica y Farmacia, Universidad Nacional de San Luis, San Luis, Argentina; ^2^Instituto de Física Aplicada (INFAP) - Centro Científico Tecnológico (CCT San Luis) - Consejo Nacional de Investigaciones Científicas y Técnicas (CONICET), San Luis, Argentina; ^3^Laboratorio de Físico-Química, Facultad de Química, Bioquímica y Farmacia, Universidad Nacional de San Luis, San Luis, Argentina; ^4^Tecnología Enzimática para Bioprocesos, Departamento de Ingeniería de Alimentos, Universidad de La Serena, La Serena, Chile; ^5^Laboratorio de Diseño y Síntesis de Péptidos, Núcleo de Biotecnología Curauma, Pontificia Universidad Católica de Valparaíso, Valparaíso, Chile; ^6^Laboratorio de Enzimas Hidrolíticas, Facultad de Ciencias, Universidad de la República (UdelaR), Montevideo, Uruguay

**Keywords:** novel antibacterial peptide, safe food preservative, peptide enzymatic synthesis, antiacanthain, granulosain, multi-point immobilization in glyoxyl-silice

## Abstract

*Antiacanthain* and *granulosain* are the partially purified proteolytic extracts from the South American native fruits of *Bromelia antiacantha* (Bertol. ) and *Solanum granuloso leprosum*, respectively. The aim of this work was to compare the ability of both soluble and immobilized *antiacanthain* and *granulosain f* or the synthesis of Z-Tyr-Val-OH, a novel antibacterial dipeptide, in different reaction systems formed by almost anhydrous organic solvents (X_w_: 1 × 10^−5^) and several percentages of immiscible organic solvents in 100 mM Tris(hydroxymethyl)aminomethane hydrochloride buffer pH 8.0. Soluble antiacanthain in half of the 24 different organic biphasic media showed higher catalytic potential than in 100 mM Tris(hydroxymethyl)aminomethane hydrolchloride buffer pH 8.0. Soluble granulosain showed lower catalytic potential in all liquid-liquid biphasic media than in the same buffer. However, 50% (v/v) ethyl ethanoate in 100 mM Tris(hydroxymethyl)aminomethane hydrolchloride buffer pH 8.0 allowed to express the highest catalytic potential of both soluble enzymes. In 50% v/v ethyl ethanoate, soluble antiacanthain and granulosain catalyzed the synthesis of Z-Tyr-Val-OH with 72 ± 0.15 and 60 ± 0.10% maximal peptide yields, respectively. Multi-point immobilization in glyoxyl-silica did not lead to better peptide yields than soluble enzymes, in that liquid-liquid biphasic medium under the same reaction conditions. Soluble and glyoxyl-silica immobilized antiacanthain in almost anhydrous ethyl ethanoate (X_w_: 1 × 10^−5^) were able to retain 17.3 and 45% of the initial proteolytic activity of antiacanthain in 100 mM Tris hydrolchloride buffer pH 8.0, respectively, at 40°C under agitation (200 rpm). Soluble and glyoxyl-silica immobilized granulosain were inactivated under the same reaction conditions. Glyoxyl-silica immobilized antiacanthain showed to be a robust biocatalyst in almost anhydrous ethyl ethanoate (X_w_: 1 × 10^−5^), eliciting the best peptide yield (75 ± 0.13%). The synthesis reaction of Z-Tyr-Val-OH could not proceed when soluble antiacanthain was used under the same conditions. Both peptidases only catalyzed the synthesis reaction under kinetic control, using activated acyl donor substrates. Finally, this work reports a novel broad-spectrum antibacterial peptide that significantly decreased (*p* ≤ 0.05) the specific growth rates of Gram positive and Gram negative microorganisms at very low concentrations (≥15 and 35 μg/ml, respectively); contributing with a new safe food preservative of applying for different food systems.

## Introduction

The demand for fresh food, easy to prepare, and ready for consumption, represents a challenge for food quality and safety. Food products can suffer undesirable microbial contamination that alter their sensory, physical-chemical and nutritional properties, causing their deterioration and significant economic losses. Furthermore, some microorganisms can cause foodborne illness (1, 2).

The growing consumer awareness of the potential negative impact of synthetic antibiotics on health has prompted the search for alternative more natural preservatives that can improve food safety and quality. Thus, several emerging biotechnologies based on bacteriophages and endolysins, inhibitors, and antagonists of bacterial quorum sensing, and antimicrobial agents from natural sources were actively researched (3–6). However, these technologies have shown several limitations such as low solubility, cytotoxicity, instability, undesirable flavors, and interactions with food components (2, 7).

In the last decades, an intense research on antimicrobial peptides (AMPs) has been reported in the literature, but only Nisin (of bacterial origin) and Lysozyme (of animal origin), called E234 and E1105 on the JECFA list of food additives, respectively; are so far the only peptides approved for commercialization by the World Health Organization (8).

The use of AMPs as food preservatives has attracted attention because they are considered as a promising and safe option in the face of the dramatic increase of microorganisms which are not only resistant to antibiotics but also are more tolerant of food processing and preservation methods (9, 10).

AMPs are quite ubiquitous conserved molecules present in all kind of organisms from prokaryotes to humans (11). In higher organisms, AMPs are components of the innate immune system (host-defense peptides) and so, beyond their direct antimicrobial activity, they are endowed with immune-modulatory properties (12). In insects and plants, AMPs act as protectants against microbial pathogens (13), while in bacteria, AMPs are used as weapons for gaining ecological niches (14). AMPs have acquired significance as new anti-infective compounds because of the rapidly increasing resistance to conventional antibiotics, so they are becoming a promising alternative to them (15). In fact, AMPs display a wide spectrum of antimicrobial activities including Gram positive and Gram negative bacteria (16), fungi (17) and viruses as targets (18), having low propensity for eliciting resistance (19, 20).

Nowadays, different technologies are available for the production of peptides: extraction from natural sources (20), recombinant DNA technology (21), production in transgenic animals and plants (22, 23) and in cell-free expression systems (24), fermentation (25), chemical synthesis (26), and enzymatic synthesis, using proteases as catalysts (27). Enzymatic synthesis of peptides using proteases offers several advantages, such as high activity, specificity and selectivity of the catalyst under moderate reaction conditions. This is usually a more economical and environmentally friendly technology than chemical processes (27). Nonetheless, some plant peptidases have been investigated as catalysts for the enzymatic synthesis of peptides (11). Particularly, the peptidases from fruits of *Bromelia antiacantha* (Bertol.) and *Solanum granuloso leprosum*, referred to as *antiacanthain* and *granulosain*, respectively, have not yet been investigated in this respect (28–33).

Vallés et al. (30) informed on a new cysteine peptidase which was purified from the *B. antiacantha* fruits, and it was named *antiacanthain A*. The partially purified fraction exhibited good specific activity from pH 5.0 to 8.0, being the highest value at pH 6.0, as well as a noticeable stability between 37and 40°C, during 3 h (30).

Vallés et al. (32) also reported on another cysteine peptidase from the *S. granuloso-leprosum* fruit; which was named granulosain I. The partially purified peptidase exhibited maximal specific activities from pH 5.2 to 8.0, and temperatures from 40 to 50°C (33).

Enzyme purification techniques show some restrictions related to the scaling up and increasing production costs to be applied to industrial process (34). Consequently, partially purified extracts were selected for this study.

Enzymatic synthesis of peptides cannot progress in aqueous solutions because the hydrolysis activity of proteases prevails over their synthesis activity. The selection of the most suitable reaction media as well as the best design and arrangement of the biocatalyst are the main requirements to achieve a successful enzymatic peptide synthesis, since the natural environment of proteases is quite different from the aggressive conditions imposed by the organic solvents (11).

The immobilization of enzymes in a certain support or matrix shows several advantages over their soluble forms, such as: greater stability of the enzyme, lower operational cost and easier separation and recovery of the enzyme for reuse (35). Furthermore, an adequate immobilization technique can improve the enzyme activity, product selectivity and substrate specificity, and reduce the enzyme inhibition by substrates or products (36).

Enzyme immobilization methods based on the formation of covalent bonds are the most widely used. The enzyme binding to the support usually involves the side chains of lysine (ϵ-amino group), cysteine (thiol group) and aspartic and glutamic acids (carboxylic group), imidazole and phenolic groups, which are not essential for the catalytic activity. The main advantage of this strategy is the stability of the bonds formed between the enzyme and the matrix, preventing the enzyme from being released into the medium (36).

The multipoint covalent immobilization of enzymes on supports activated with epoxy groups can be a very useful tool to stabilize industrial enzyme. Silica matrix has a tunable surface comprising hydrophilic silanol groups and moderately hydrophobic siloxane groups, which can be modified by chemical treatments with organic functional groups to achieve a robust biocatalyst. Glyoxyl-silica is a monofunctional matrix that forms multi-point covalent linkages with non-ionized primary amines and lead to stable bonds after a final reduction step (37). An original contribution of this paper is the immobilization of antiacanthain and granulosain in a glyoxyl-silica support. As far as is known, there are no other works reported on the aforementioned immobilized enzymes or on their application to peptide synthesis.

The aim of this work was to compare the ability of both soluble and immobilized antiacanthain and granulosain for the synthesis of N-α-carbobenzyloxy-L-Tyrosyl-L-Valine (Z-Tyr-Val-OH), a novel antibacterial dipeptide, in different reaction systems formed by almost anhydrous organic solvents (X_w_: 1 × 10^−5^), and several percentages (30, 50, and 70% v/v) of immiscible organic solvents in 100 mM Tris(hydroxymethyl)aminomethane hydrochloride buffer pH 8.0.

## Materials and Methods

### Reagents

(3-Glycidoxypropyl)trimethoxysilane (GLYMO, ≥99%), Octyltriethoxysilane (OTEOS, 97.5%), cetyltrimethylammonium bromide (CTAB, ≥99%), N-α-benzoyl-DL-arginine-p-nitroanilide (DL-BAPA, ≥96.0% high quality grade for HPLC), monobasic sodium orthophosphate (p.a., anhydrous, ≥99.0%), silicon dioxide (~99%, 0.5–10 μm), ethyl ethanoate (99%), sodium tetrahydridoborate (99%), Tris(hydroxymethyl)amino methane hydrochloride (≥99%), tetraethylammonium chloride [BioUltra, for molecular biology (≥99% AT)], L-Valine (Val-OH), Z-Tyr(Bzl)-OH (TLC, ≥98%), N-alpha-carbobenzoxy-L-amino acid 4-nitrophenyl ester hydrochloride (98%), 2-hydroxyethylmercaptan (≥99%), trifluoroethanoic acid (≥99%), 4-nitroaniline (≥99.0%), 4-nitrophenol (≥99%), bovine albumin (≥96%), and 1,2,3-propanetriol (99%) were bought to Sigma-Aldrich (St. Louis, MO, USA). Sodium periodate (≥99.5%), sodium trisilicate solution (≥99%), sodium hydrogen carbonate (99.7%), L-cysteine hydrochloride hydrate (≥99%), phosphate buffer (pH 7.2) and carbonate-bicarbonate buffer (pH 10), diaminoethane-tetraacetic acid (90%), sulfuric acid (99%), and sodium hydroxide solution (≥99%) were bought to Merck KGaA (Darmstadt, Germany). Dimethyl sulfoxide (for headspace gas chromatography SupraSolv®), acetonitrile, acetone, benzene, octanol, dichloromethane, diethyl ether, cyclohexane, chlorobenzene, acetophenone, and ethyl ethanoate (gradient grade for liquid chromatography LiChrosolv®), formic acid (98–100% for HPLC LiChropur™) were also bought in Merck KGaA (Darmstadt, Germany).

### Plant Material

Samples of plant material were deposited in the Uruguayan Botanical Garden Museum “Prof. Atilio Lombardo,” which is part of the Municipality of Montevideo, Uruguay. The samples of these species were cataloged and inventoried by the Museum as N° MVJB-23895 for *Bromelia antiacantha* and N° MVJB-9276 for *Solanum granuloso-leprosum*.

### Preparation of Partially Purified Enzymatic Extracts

Antiacanthain and granulosain, the partially purified proteolytic extracts from the native ripe fruits of *Bromelia antiacantha* (Bertol.) and *Solanum granuloso leprosum*, respectively, were obtained according to Vallés et al. (31–33); and lyophilized for later immobilization in glyoxyl-silica supports.

### Electrophoresis

The molecular size (MW) of the proteins was determined by denaturing and reducing electrophoresis in a TRICINE SDS-PAGE differential buffer system, described by Schagger and Von Jagow (38). Electrophoresis was carried out at constant voltage (30 V) until the samples reached the end of the concentrating gel. The voltage was progressively increased every 10 s as the proteins entered the spacer gel until reaching 100 V, a value that was kept constant until the end of the electrophoresis.

### Protein Concentration and Enzyme Specific Activity Assays

Proteins concentration was determined by Bradford protein assay, with bovine albumin as protein standard (39). The proteolytic specific activity of antiacanthain (0.075 mg of protein/ml) and granulosain (0.125 mg of protein/ml) was measured with DL-BAPA as substrate. Briefly, 0.5 ml of partially purified peptidase was incubated with 0.5 ml of 5 mM DL-BAPA and 20 mM L-cysteine in 100 mM Tris(hydroxymethyl)amino methane hydrochloride buffer pH 8 for 5 min at 37°C, under agitation at 200 rpm. The absorbance of the reaction product was measured using a spectrophotometer (UV-Vis Spectrometer, Cintra 4040, GBC Scientific Equipment Ltd., Hampshire IL, USA) at λ: 410 nm. Product quantification was performed using a standard curve of p-nitroaniline in 100 mM Tris(hydroxymethyl)aminomethane hydrochloride buffer pH 8.0.

The enzymatic activity was expressed as international units (IU). One IU was defined as the amount of peptidase (antiacanthain or granulosain) that releases 1 μmol of p-nitroaniline per min under the conditions mentioned above. As a negative control, the activity assay was performed with buffer solution instead of enzymatic extracts.

### Stability of Proteolytic Enzymes

The stability of anticanthain and granulosain, in solution or covalently linked to glyoxyl-silica support, was assayed under non-reactive conditions at 40°C under agitation (200 rpm) in: 100 mM Tris(hydroxymethyl)amino methane hydrochloride buffer pH 8, liquid-liquid biphasic systems constituted by 30, 50, and 70% v/v of hydrophobic organic dissolvent in 100 mM Tris(hydroxymethyl)amino methane hydrochloride buffer pH 8.0, and in almost anhydrous organic solvents (X_w_: 1 × 10^−5^). Immiscible organic solvents were selected from a design of experiments carried out with 72 organic solvents, which clustered them with respect to their physicochemical characteristics (40).

The residual activity (%) within a time range, which was considered a sound criterion for enzyme replenishment, was measured as detailed below. The catalytic potential of each peptidase (CP) was evaluated from the area integration under the enzyme inactivation curves (Equation 1).

(1) $$\begin{array}{r} \text{CP}=\overset{\text{tf}}{\underset{0}{\int }}\text{A}\cdot \text{dt} \end{array}$$

Where, A is the expressed activity from initial to a final replenishment time (t_f_) under stability conditions. CP is a helpful descriptor for comparing the performances of several biocatalysts, because it involves both the enzyme activity and enzyme stability (41).

### Determination of Water Content of the Enzymes in Almost Anhydrous Organic Solvents

The effect of different organic solvents on the stability of antiacanthain and granulosain in almost anhydrous organic solvents was studied at low and constant water content in the enzymes. The amount of water on the enzyme after incubation in each organic solvent with different concentration of water was determined as follows: 0.4 g of lyophilized enzyme and 1 ml of each organic solvent with different degree of hydration were suspended and incubated for 10 min at 40°C in a previously weighted centrifuge tube. The suspension was centrifuged at 6,000 rpm (Refrigerated Centrifuge, Model 5430R Eppendorf, Medical Equipment Specialists Inc., Ma, USA) for 5 min and the amount of water in the supernatant and in the pellet was determined using a Karl Fisher Coulomb Titrator (model CA-200, Mitsubishi).

Total amount of water in the pellet is the sum of water bound to the enzyme and the amount of water into the organic solvent trapped in the enzyme. Then, the net amount of water bound to the enzyme was determined by subtracting the amount of water bound to the trapped organic solvent to the amount of water in the pellet. The amount of trapped organic solvent was calculated on the basis of the weight of the entrapped organic solvent (difference between the weight of the tube with the pellet and the weight of the tube with the dry enzyme) and the amount of water in the solvent (supernatant) (42).

The water content in the organic solvent and in the enzyme was determined as volume percentage (% v/v) and weight percentage (% w/w). The amount of water in the enzyme was expressed as relative fraction of water (X_w_), which is the ratio between the molar concentration of water in the enzyme and the molar concentration of pure water, and this value was set at X_w_: 1 × 10^−5^ (Equation 2).

(2) $$\begin{array}{r} {\text{X}}_{\text{w}}=\frac{\text{Concentration of water in the enzyme }\left(\text{M}\right)}{\text{Pure total water concentration }\left(55\text{M}\right)} \end{array}$$

### Enzyme Preferences for Synthetic Substrates

Preferences for peptidase substrates were evaluated as esterolytic activity, using N-alpha-[(benzyloxy)carbonyl]-L-amino acid-p-nitrophenyl esters as synthetic substrates (43). The trials were carried out with 0.05 ml of enzymatic solution, 2 mM diaminoethane-tetraacetic acid and 20 mM L-cysteine, 0.1 ml of 1 M substrate (previously solubilized in dimethyl sulfoxide) and 0.85 ml of 50% v/v ethyl ethanoate in 100 mM diaminoethane-tetraacetic acid buffer pH 8. The reactants were placed in a water bath at 40°C during 5 min and the liberated 4-nitrophenol was measured at λ: 405 nm. A calibration curve of 4-nitrophenol allowed to evaluate the liberated p-nitrophenolate. Besides, controls in absence of substrates or peptidases were performed. One unit of esterolytic activity (U_CBZ_) was defined as the quantity of protease necessary to liberate 1.0 μmol of 4-nitrophenol per min under the described assay conditions.

### Immobilization by Multi-Point Covalent Binding of Anticanthain and Granulosain in Glyoxyl-Silica

Initially, a reaction mixture containing the following proportional composition was prepared: 1 SiO_2_: 0.30 Na_2_O: 0.24 hexadecyltrimethylammonium bromide (CTAB): 7.2 ethyl ethanoate: 193 H_2_O. Then, for the synthesis of silica, 1.6 g CTAB (4.4 mm) was dissolved in 60 ml of water (in order to obtain a concentration of CTAB above its critical micelle concentration); 12.8 ml ethyl ethanoate was added to the surfactant solution and 4 g of sodium silicate (18 mm) was dispersed by magnetic stirring. The mixture was heated at 80°C for 48 h without stirring. The solid obtained was incinerated at 540°C (heating rate: 1.58°C/min) in a muffle furnace (Thermo Scientific Thermolyne FA48020-33, IL, USA) for 3 h. The synthesized silica was chemically modified as follows: 1 g of silica (activated under vacuum at 200°C) was silylated at 94°C in 30 ml of 10% v/v aqueous solution of glycidyl oxypropyl trimethoxysilane (GPTMS) (pH 8.5), during 6 h under gentle agitation. The hydrolysis of epoxy groups was carried out with H_2_SO_4_ (0.1 M) at 85°C, during 2 h. After filtration, the support was washed with water/acetone mixture (70:30) and dried. The oxidation was carried out by contacting the support with 0.1 M NaIO_4_ at 25°C, during 2 h. The concentration of glyoxyl groups was quantified by back titration with NaHCO_3_/KI, using a spectrophotometer (UV-Vis Spectrometer, Cintra 4040, GBC Scientific Equipment Ltd., Hampshire IL, USA) at λ: 420 nm (44).

The support (0.309 g) was contacted with a solution of antiacanthain or granulosain previously prepared in 0.1 M NaHCO_3_ buffer pH 10, at 25°C, during 2 h, under stirring at 200 rpm. 1 ml of supernatant was taken for determining enzyme activity. An immobilization control (soluble enzyme) and a reagent control (without DL-BAPA) were also carried out. The biocatalyst was reduced using NaBH_4_ (1 mg/ml of enzyme solution), during 15 min at 4°C, under agitation at 200 rpm. Finally, the support was washed with distilled water. The immobilization yield in terms of activity (Ya) was calculated as the percentage of the ratio between the offered activity (A_o_) and the expressed activity in the biocatalyst (A), according to Equation (3):

(3) $$\begin{array}{r} \text{Ya}=\frac{\text{A}}{{\text{A}}_{0}}\cdot 100 \end{array}$$

### Enzyme Synthesis of Z-Tyr-Val-OH Dipeptide

The enzymatic synthesis of Z-Tyr-Val-OH was carried out under kinetic and thermodynamic control, using solubilized, and glyoxyl-silica immobilized peptidase (antiacanthain or granulosain), in a liquid-liquid biphasic system (50% v/v ethyl ethanoate in 100 mM Tris(hydroxymethyl)amino methane hydrochloride buffer pH 8) and in almost anhydrous organic solvent (ethyl ethanoate, X_w_: 1 × 10^−5^), at 40°C under agitation (200 rpm).

The selection of N-alpha-[(benzyloxy)carbonyl]-L-Tyr-p-nitrophenyl ester (Z-Tyr-pNO) and L-valine (Val-OH), as acyl donor and nucleophile substrates, respectively, was made from the enzyme preference tests.

The amount of Z-Tyr-pNO was determined from its maximum solubility in the immiscible organic dissolvent of the liquid-liquid medium, the partition coefficient between the phases, and the kinetic parameters (*K*_*m*_) of anticanthain and granulosain.

The reaction of synthesis under kinetic control was carried out in a liquid-liquid biphasic system, consisting of an aqueous phase (100 mM Tris(hydroxymethyl)aminomethane hydrochloride buffer pH 8.0) containing 0.075 mg/ml of soluble antiacanthain (1.875 IU/ml) or 0.125 mg/ml of soluble granulosain (1.850 IU/ml), 20 mM 2-mercaptoethanol, 7.55 mM of both Val-OH and triethyl ammonium (TEA), and an organic phase (50% v/v ethyl ethanoate) containing 17.25 mM of Z-Tyr-pNO. The reaction was conducted at 40°C in a GFL Shaking Incubator Orbital Motion (Model 3031, Germany) at 200 rpm. Aliquots (0.1 ml) were taken from the aqueous and organic phase at different times during 24 h and mixed with 0.1 ml of 1% v/v trifluoroacetic acid (TFA) for quenching the reaction. In addition, the synthesis reaction under thermodynamic control was also carried out using 17.25 mM Z-Tyr-OH as acyl donor (instead of Z-Tyr-pNO), under the same reaction conditions.

On the other hand, the synthesis of Z-Tyr-Val-OH under kinetic and thermodynamic control was studied using the peptidases suspended in almost anhydrous ethyl ethanoate (X_w_: 1 × 10^−5^ in the enzyme), containing 17.25 mM of Z-Tyr-pNO (or Z-Tyr-OH), 20 mM 2-mercaptoethanol, and 17.55 mM of both Val-OH and TEA.

In all the synthesis reactions described above, antiacanthain (12.5 mg/ml) and granulosain (20.2 mg/ml) immobilized on glyoxyl silica were used. Besides, the acyl acceptor concentration was increased until its maximum solubility (75.5 mM) in order to maximize peptide yield (η).

Samples (100 μl) were periodically withdrawn during 48 h from the reaction mixture supernatant, and 0.1 ml of 1% v/v TFA were added for quenching the reaction. The reaction components were analyzed by RP-HPLC, and the synthesized peptide was purified by chromatography, using a SPE-Cartridge C_18_ (RP18, ODS, Octadecyl, 3 ml tube size, 500 mg sorbent weight) (Merck, Darmstadt, Germany), lyophilized and identified by mass spectrometry (MS). At the same time, several blanks were carried out using the protease only in the aqueous-organic medium, each substrate separately and all reactants in absence of enzyme.

Peptide yield (η) and conversion of acyl donor substrate (α_s_) (45) were determined according to Equations (4, 5):

(4) $$\begin{array}{r} \eta =\frac{\left[P\right]}{\left[S_{0}\right]}100 \end{array}$$

(5) $$\begin{array}{r} \alpha_{s}=\frac{\left[S_{0}-{\text{S}}_{\text{t}}\right]}{{\text{S}}_{0}}100 \end{array}$$

Where, S_o_ is the amount of acyl donor substrate at the initial time of reaction, S_t_ is the amount of acyl donor substrate at a particular time of reaction and P is the amount of synthetized peptide.

### Analytical Control of Peptide Synthesis

#### RP-HPLC

Reactions were monitored by RP-HPLC (Gilson, Model 712, Middleton, WI, USA) using a C_18_ column, 4.60 × 250 mM (Phenomenex, Torrance, CA, USA) and UV Detector at λ: 254 nm and 25°C. The injection volume was 20 μl, the flow rate of the mobile phase (50% v/v methyl cyanide in Tris(hydroxymethyl)amino methane hydrochloride buffer pH 8) was 800 μl/min. The product was purified by means of a C_18_ cartridge (Merck) (>95% purity) and dried in a concentrating device (Thermo Scientific Savant™ SPD131DDA SpeedVac, Madrid, España). The purified peptide was analyzed on a High-performance liquid chromatography (Jasco, PW de Meern, Nederland) equipped with a Photo Diode Array Detector, using a XBridge™ BEH C_18_ column (100 × 4.6 mm, 3.5 μm) (Waters Corporation). The operating conditions were: injection volume of 20 μl, flow rate of 1 ml/min, mobile phase formed by A: Mili Q water with 2.5% fluoroacetic acid; and 30–100% gradient of B: methyl cyanide with 2.5% fluoroacetic acid for 20 min).

#### Mass Spectrometry

The ESI-MS analysis of peptides was carried out with a Shimadzu LC-MS 2020 (Montevideo, Uruguay), using 2 μg of peptide. The mass spectrometer was optimized for 70–3000 m/z, with a 4.5 kV positive polarity, a 4500 V capillary voltage, 8.0 psi nebulizer gas, 5.0 L/min dry gas, heater interface temperature of 350°C, for 30 min. The data were processed by means of Shimadzu Software (LabSolution, Version 5.42).

#### Matrix-Assisted Laser Desorption/Ionization Time-of-Flight Mass Spectrometry

The desalted peptides were mixed with a 1:1 (v/v) mixture of α-cyano-4-hydroxy cinnamic acid (HCCA) saturated with a solution comprising 30% methyl cyanide and 0.1% methanoic acid, applied to a polished steel plate, and subjected to mass characterization by a Microflex® MALDI-TOF mass spectrometer (Bruker Daltonics, Bremen, Germany), which was operated in positive ion mode and reflector detector, and previously calibrated with an external standard (700–1800 Da). The data were processed by means of the FlexControl Software (Version 3.0, Bruker Daltonics GmbH).

### Bacterial Strains

Eight Gram positive and Gram negative strains were assayed in this work. *Staphylococcus aureus* ATCC 25923 and *Escherichia coli* ATCC 25922 (American Type Culture Collection, Manassas, VA, USA) were used as Gram positive and Gram negative reference strains, respectively.

Besides, six wild strains isolated in the Central Microbiology Laboratory of the San Luis Ministry of Health, Government of San Luis, Province of San Luis, Argentina, were also used. They were identified as:

- *Staphylococcus aureus* C00195 (Gram positive bacterium, sensitive to commonly used antibiotics, such as methicillin, penicillin, gentamicin, erythromycin, ciprofloxacin);
- *Staphylococcus hominis* A17771 (Gram positive bacterium, sensitive to commonly used antibiotics, such as penicillin and erythromycin);
- *Enterococcus faecalis* I00125 (Gram positive bacterium with multidrug resistance to commonly used antibiotics, such as vancomycin and β-lactam antibiotics);
- *Escherichia coli* A17683 (Gram negative bacterium, sensitive to commonly used antibiotics).
- *Klebsiella oxytoca* A19438 (Gram negative bacterium with natural or intrinsic resistance to ampicillin).
- *Pseudomonas aeruginosa* C00213 (Gram negative bacterium resistant to carbapenem antibiotics).

Bacterial strains were grown for 24 h at 37°C on Müller-Hinton agar (Laboratorios Britania S.A., Buenos Aires, Argentina) and they were maintained by successive peals.

### Antibacterial Activity of Z-Tyr-Val-OH

The antibacterial activity of Z-Tyr-Val-OH against both Gram positive and Gram negative bacteria was assayed by a kinetic-turbidimetric method which was described by Talia et al. (46–48). The cultures were aseptically transferred to 30 ml of a Müller-Hinton broth and incubated at 37°C for 18 h with gentle agitation, to be used as inoculum. The kinetic assays of microbial growth were carried out in 10 ml Erlenmeyer flasks with Müller-Hinton broth containing 0.2 ml of inoculum, without peptide (control) and with increasing peptide concentrations (samples). Erlenmeyer flasks were incubated in a GFL Shaking Incubator Orbital Motion (Model 3031, Burgwedel, Germany) at 40°C, under agitation at 180 rpm. Aliquots were periodically withdrawn at 20 min intervals for 5 h and the transmittance (T) was read at λ: 720 nm. The transmittance (T) was related to *Nt* (Colony Forming Units (CFU)/ ml) in order to apply the following equations:

(6) $$\begin{array}{r} \text{ln}Nt\text{ Gram positive}=27.4-10.3\cdot T \end{array}$$

(7) $$\begin{array}{r} \text{ln}Nt\text{ Gram negative}=27.1-8.56\cdot T \end{array}$$

Specific growth rates of bacteria were determined from ln *Nt* vs. time plots. Minimum inhibitory concentrations (MICs) of Z-Tyr-Val-OH against both Gram positive and Gram negative bacteria were calculated from the plots of specific growth rate vs. peptide concentration. MIC is defined as the lowest concentration of an antimicrobial that will inhibit the visible growth of a microorganism after overnight incubation. The results obtained were compared with oxacillin (a second generation penicillin antibiotic against moderate-to-severe penicillinase-resistant staphylococcal infections), and nalidixic acid (a synthetic naphthyridone antibiotic against Gram negative bacteria such as *E. coli*) (46–48).

### Data Statistical Analysis

Residual enzyme activity, activity yield (Ya), degree of conversion of substrate (α_s_) and peptide yield (η) were calculated from three separately assays which were performed in duplicate and the results were informed as mean ± SD. The linear region of the reaction progress of enzyme activity was also determined. Antimicrobial activity was also obtained from three independent trials which were done by duplicate, and data were reported as mean ± SD. The Kruskal-Wallis method was used to test the significant differences (*p* ≤ 0.05) between Z-Tyr-Val-OH antimicrobial activity at different concentrations and the control assay. The Kruskal-Wallis test was selected because it is a non-parametric test that is applied to more than two independent samples, with a reduced number of repetitions (*N* < 50) (49). The data were processed by means of the IBM® SPSS® Statistics V22.0 Software.

## Results and Discussion

### Antiacanthain and Granulosain, Partially Purified Peptidases From Native Fruits

Antiacanthain and granulosain, the partially purified proteolytic extracts from the native ripe fruits of *Bromelia antiacantha* (Bertol.) and *Solanum granuloso leprosum*, respectively, were obtained using a low-cost purification method based on the protein acetone precipitation (31–33).

**Figure 1:**
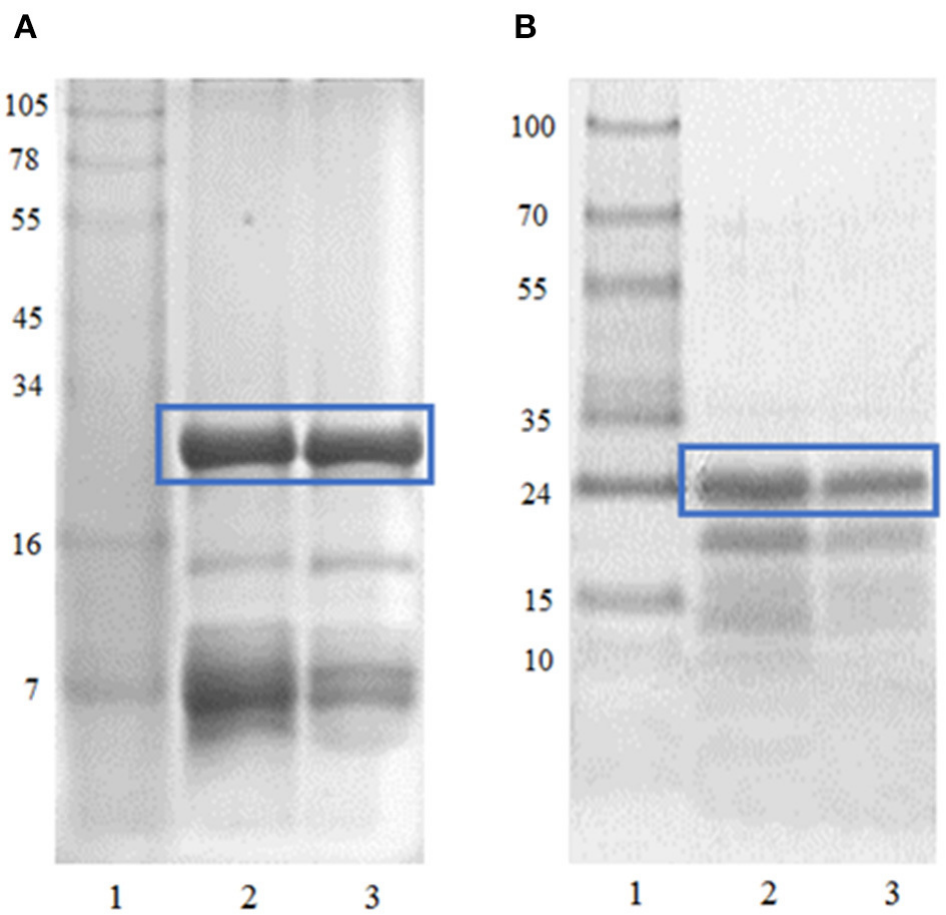
**(A)** Tricine SDS-PAGE. Lane 1: MW Maker See-blue (Invitrogen), Lane 2: *Bromelia antiacantha* crude extract, Lane 3: Antiacanthain. **(B)** Tricine SDS-PAGE. Lane 1: MW Protein marker V (Avantor), Lane 2: *Solanum granuloso-leprosum* crude extract, Lane 3: Granulosain.

The crude extracts of *B. antiacantha* and *S. granuloso leprosum* fruits contain high concentrations of carbohydrates, pigments, phenolic compounds, and vitamin C (50, 51). Furthermore, these crude extracts contain several times more protein than the partially purified extracts used in this work. As shown in Figures 1A,B, a high purification degree of the main protein fractions was achieved, corresponding to antiacantain (23.335 kDa) and granulosain (23.878 kDa), respectively. Both fractions showed high specific activity in 100 mM Tris (hydroxymethyl)amino methane hydrochloride buffer pH 8, being 25 IU/mg of protein for antiacantain and 14.8 IU/mg of protein for granulosain.

### Immobilization by Multi-Point Covalent Binding of Antiacanthain and Granulosain in a Glyoxyl-Silica Carrier

In order to obtain robust biocatalysts able to withstand the aggressive reaction conditions of the peptide synthesis, antiacanthain, and granulosain were immobilized in glyoxyl-silica carrier by means of multi-point covalent bonding. Silica was the selected support material because it is one of the most appropriate materials for forming multi-point Schiff base bonds (52).

The offered activity of antiacanthain was 49 ± 0.01 IU/ml, equivalent to 0.32 ± 0.02 IU/mg of support. After immobilization, the remaining activity in the supernatant was 14 ± 0.01 IU/ml, equivalent to 0.092 ± 0.01 IU/mg of support. The difference between them was expressed as theoretical activity. The activity of immobilized antiacanthain in glyoxyl-silica was 0.15 ± 0.01 IU/mg of support, and Ya was 47 ± 0.01%. The offered activity of granulosain was 50 IU/ml, equivalent to 0.33 ± 0.02 IU/mg of support. After immobilization, the remaining activity in the supernatant was 20.7 ± 0.01 IU/ml, equivalent to 0.138 ± 0.01 IU/mg of support. The activity of immobilized granulosain in glyoxyl-silica was 0.093 ± 0.01 IU/mg of support, and Ya was 28.18 ± 0.01%.

A higher Ya was obtained for antiacanthain under the studied conditions. However, both enzymes showed 2.1–3.5 times lower expressed activity than the offered activity. It is likely that during the immobilization process the Lys groups of the microenvironment of the active site have bound to the support, hampering or decreasing the specific activity of antiacanthain and granulosain at pH 10 (30–33).

### Catalytic Potential of Soluble and Immobilized Antiacanthain and Granulosain

Antiacanthain and granulosain catalytic potentials were studied under non-reactive conditions, at 40°C under agitation at 220 rpm, in several systems: 100 mM Tris(hydroxymethyl)amino methane hydrochloride buffer pH 8, liquid-liquid biphasic systems (30, 50, and 70% v/v organic solvent in the same buffer), and almost anhydrous organic solvents (X_w_: 1 × 10^−5^).

The proteolytic activity and the operational stability of soluble and glyoxyl-silica immobilized enzymes were evaluated using the catalytic potential as criteria (Equation 1) in order to select the most robust catalyst and the most promissory medium for the synthesis of Z-Tyr-Val-OH.

As shown in Table 1, soluble antiacanthain in half of the 24 different aqueous-organic biphasic media studied showed higher catalytic potential than in 100 mM Tris(hydroxymethyl)amino methane hydrochloride buffer pH 8. The high stability shown by antiacanthain is because the enzyme is dissolved in the aqueous phase in the immiscible liquid-liquid systems, and it does not come into contact with the organic solvent (40, 53). Besides, the catalytic potential of antiacanthain was independent of the organic solvent concentration, indicating that the concentration did not alter the protein-water interactions in the enzyme's surrounding microenvironment and maintained its active structure. This behavior, named molecular toxicity, is well-known for other proteolytic enzymes (45, 54).

**Table 1:** Catalytic potential of soluble antiacanthain (0.075 mg/ml, 1.875 IU/ml) after 6 h in liquid-liquid biphasic systems constituted by several concentrations of organic solvent in 100 mM Tris(hydroxymethyl)amino methane hydrochloride buffer pH 8, at 40°C.

**Organic solvent**	**Catalytic potential (IU/mg** **·** h)^*^		
	**30%**	**50%**	**70%**
Benzene	140.70 ± 0.01^h^	125.33 ± 0.19^f^	121.75 ± 0.05^g^
Octanol	114.19 ± 0.25^e^	108.03 ± 0.05^c^	78.33 ± 0.01^b^
Dichloromethane	123.64 ± 0.01^f^	116.77 ± 0.05^d^	104.39 ± 0.10^d^
Diethyl ether	97.68 ± 0.05^c^	93.07 ± 0.09^b^	116.71 ± 0.34^e^
Cyclohexane	134.71 ± 0.05^g^	147.19 ± 0.01^g^	120.82 ± 0.05^f^
Chlorobenzene	68.76 ± 0.23^a^	123.57 ± 0.01^e^	56.50 ± 0.02^a^
Acetophenone	85.57 ± 0.01^b^	89.22 ± 0.01^a^	83.24 ± 0.01^c^
Ethyl ethanoate	104.97 ± 0.15^d^	170.62 ± 0.01^h^	126.40 ± 0.01^h^

*The catalytic potential of antiacanthain in 100 mM Tris(hydroxymethyl)amino methane hydrochloride buffer pH 8, at 40°C was 116.48 ± 0.01 (IU/mg · h)*.

* *Catalytic potential data are expressed as the mean ± SD (standard deviation) of three independent experiments which were done in duplicate. The values with different superscript letters in a column are significantly different (p < 0.05) (IBM SPSS Statistics 27.0)*.

Unlike antiacanthain, soluble granulosain showed lower catalytic potential in all liquid-liquid biphasic media than in 100 mM Tris(hydroxymethyl)amino methane hydrochloride buffer pH 8 (Table 2). The partitioned organic solvent into aqueous phase affected the catalytic expression of granulosain, as a consequence of the changes that the secondary structure of granulosain underwent in those media (55). Besides, the increased interface when using organic solvent at 70% (v/v) in the biphasic systems seems to be the cause of the decrease or loss of enzyme activity. This behavior has been named phase toxicity in the literature (56).

**Table 2:** Catalytic potential of soluble granulosain (0.125 mg/ml, 1.850 IU/ml) after 6 h in liquid-liquid biphasic systems constituted by several concentrations of organic solvent in 100 mM Tris(hydroxymethyl)amino methane hydrochloride buffer pH 8, at 40°C.

**Organic solvent**	**Catalytic potential (IU/mg** **·** h)^*^		
	**30%**	**50%**	**70%**
Benzene	76.38 ± 0.15^a^	139.42 ± 0.00^d^	56.09 ± 0.24^d^
Octanol	13.05 ± 0.09^e^	17.92 ± 0.01^a^	0 ± 0.00^a^
Dichloromethane	121.57 ± 0.01^h^	191.09 ± 0.01^g^	29.98 ± 0.33^c^
Diethyl ether	38.53 ± 0.00^c^	25.47 ± 0.05^b^	0 ± 0.00^a^
Cyclohexane	92.31 ± 0.28^f^	176.92 ± 0.03^e^	57.90 ± 0.25^e^
Chlorobenzene	73.18 ± 0.01^d^	188.01 ± 0.01^f^	61.15 ± 0.01^f^
Acetophenone	16.45 ± 0.05^b^	64.77 ± 0.20^c^	0 ± 0.00^a^
Ethyl ethanoate	117.56 ± 0.01^g^	200.14 ± 0.01^h^	4.42 ± 0.00^b^

*The catalytic potential of granulosain in 100 mM Tris(hydroxymethyl)amino methane hydrochloride buffer pH 8, at 40°C was 350 ± 0.01 (IU/mg · h)*.

* *Catalytic potential data are expressed as the mean ± SD (standard deviation) of three independent experiments which were done in duplicate. The values with different superscript letters in a column are significantly different (p < 0.05) (IBM SPSS Statistics 27.0)*.

However, 50% v/v ethyl ethanoate in 100 mM Tris(hydroxymethyl)aminomethane hydrochloride buffer pH 8.0 allowed to express the highest catalytic potential of both soluble peptidases (antiacanthain and granulosain). For this reason, this liquid-liquid biphasic medium was selected for the synthesis of Z-Tyr-Val-OH, using soluble and immobilized enzymes. Glyoxyl-silica immobilized peptidases showed significant lower catalytic potential than soluble peptidases in that biphasic medium.

Furthermore, almost anhydrous organic solvents were prepared with the addition of a certain water concentration in order to obtain a constant relative fraction of water (X_w_) in the enzymes of 1 × 10^−5^. A constant relative fraction of water higher than 1 × 10^−5^ led to the formation of biphasic systems when some of the organic solvents were used.

**Figure 2:**
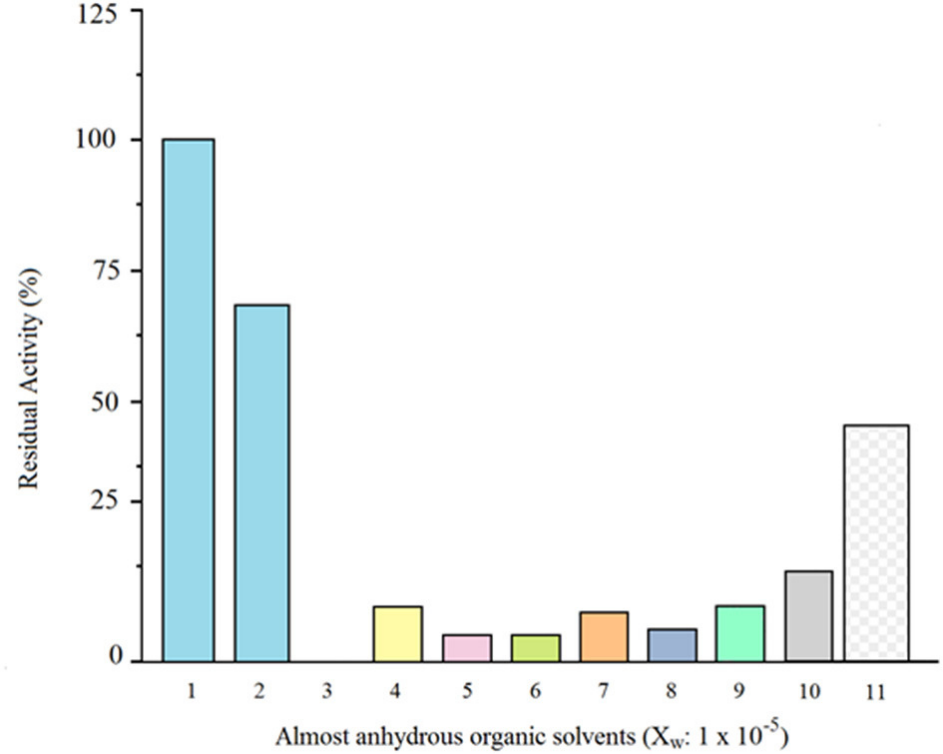
Comparative residual activity (%) of soluble antiacanthain (0.075 mg/ml, 1.875 IU/ml) in 100 mM Tris(hydroxymethyl)amino methane hydrochloride buffer pH 8 vs. soluble antiacanthain (0.075 mg/ml) in almost anhydrous organic solvents (X_w_: 1 × 10^−5^) and glyxoyl-silica immobilized antiacanthain (12.5 mg/ml) in almost anhydrous ethyl ethanoate (X_w_: 1 × 10^−5^), after 6 h of incubation at 40°C under agitation at 200 rpm. (1) Control (Tris buffer pH 8, initial specific activity: 100%); (2) Tris buffer pH 8; (3) Diethyl ether; (4) Chlorobenzene; (5) Acetophenone; (6) Octanol; (7) Benzene; (8) Dichloromethane; (9) Cyclohexane; (10) Ethyl ethanoate (soluble antiacanthain); (11) Ethyl ethanoate (glyoxyl-silica immobilized antiacanthain).

Soluble and glyoxyl-silica immobilized granulosain were completely inactivated in almost anhydrous organic solvents. Under these conditions, the soluble and immobilized antiacanthain showed low activity, but after 6 h in ethyl ethanoate (X_w_: 1 × 10^−5^) they were able to retain 17.3 and 45% of the initial specific activity in 100 mM Tris hydrochloride buffer pH 8.0 at 40°C under agitation (200 rpm), respectively (Figure 2). For this reason, ethyl ethanoate (X_w_: 1 × 10^−5^) was selected as the almost anhydrous organic medium for the synthesis of Z-Tyr-Val-OH, using soluble and immobilized antiacanthain.

### Antiacanthain and Granulosain Preferences

The amino acid side chain parameters play an important role in the specificity of the enzyme because they are responsible for the hydrophobic, steric, and electronic interactions in the surroundings of the active site (43).

Table 3 shows the specificity of antiacanthain and granulosain using different synthetic amino acid substrates in 50% v/v ethyl ethanoate in 100 mM Tris (hydroxymethyl)aminomethane hydrochloride buffer pH 8.0, at 40°C. Antiacanthain showed high preference for polar substrates, such as the synthetic derivatives of Asn, Tyr, Gln, and Val. Granulosain in 50% v/v ethyl ethanoate in 100 mM Tris(hydroxymethyl)aminomethane hydrochloride buffer pH 8.0 showed high preferences for Tyr, Val, Ile, and Asp.

**Table 3:** Preferences of the partially purified enzymatic extracts: soluble antiacanthain (0.075 mg/ml, 1.875 IU/ml) and soluble granulosain (0.125 mg/ml, 1.850 IU/ml), for N-alpha-[(benzyloxy)carbonyl]-amino acid-4-nitrophenyl esters, at 40°C.

**N-alpha-[(benzyloxy)carbonyl]-amino acid-4-nitrophenyl esters**	**Antiacanthain**		**Granulosain**	
	**U_**CBZ**_**	**Preference (%)**	**U_**CBZ**_**	**Preference (%)**
Tyr	47.95 ± 0.05^j^	90.6	2.20 ± 0.147^c^	100
Asn	52.91 ± 0.07^k^	100	1.90 ± 0.121^bc^	86.4
Gln	41.40 ± 0.40^i^	78.2	1.60 ± 0.170^b^	72.7
Gly	35.08 ± 0.42^g^	66.3	1.60 ± 0.103^b^	72.7
Lys	31.9 ± 0.085^f^	60.3	0.00 ± 0.00^a^	0
Phe	0.00 ± 0.00^a^	0	1.00 ± 0.125^d^	45.5
Trp	5.94 ± 0.06^b^	11.2	0.30 ± 0.147^a^	13.6
Val	35.77 ± 0.22^h^	67.6	2.10 ± 0.193^c^	95.5
Ile	12.87 ± 0.12^e^	24.3	2.00 ± 0.147^bc^	90.9
Leu	8.86 ± 0.14^c^	16.7	0.00 ± 0.00^a^	0
Ala	11.95 ± 0.05^d^	22.6	0.00 ± 0.00^a^	0

*The values with different superscript letters in a column are significantly different (p < 0.05) (IBM SPSS Statistics 27.0)*.

In peptide synthesis by protease-mediated reactions, the acyl donor binds to the S subsite of the enzyme and the amino component that acts as an acyl acceptor interacts with the S' binding site (57). The reaction rate will depend on the acyl donor specificity by the protease. The efficiency of the nucleophilic attack of the added amino component depends on an optimal binding in the region of the S' subsite of the protease, being crucial for obtaining a high peptide yield.

Antiacanthain and granulosain showed high preference for the Tyr derivative, using *p*-nitrophenyl esters of *N*-protected amino acids as substrates, suggesting that Z-Tyr-pNO would be a suitable acyl donor substrate for the synthesis reaction. Then, Z-Tyr-pNO and Val-OH were chosen as acyl donor and acyl acceptor, respectively, for the synthesis of Z-Tyr-Val-OH.

### Enzymatic Synthesis of Peptide

The kinetically-controlled synthesis of Z-Tyr-Val-OH was carried out with Z-Tyr-pNO and Val-OH as acyl donor and acyl acceptor, respectively, in two different reaction media formed by 50% v/v ethyl ethanoate in 100 mM Tris(hydroxymethyl)amino methane hydrochloride buffer pH 8.0, and almost anhydrous ethyl ethanoate (X_w_: 1 × 10^−5^ in the enzymes into the organic solvent). Those media allowed a high solubility of the substrates, which were 75.5 mM for Val-OH in water and 189 mM for Z-Tyr-pNO in ethyl ethanoate, an acyl donor partition coefficient of 1.28 and the best catalytic potential of both enzymes (Tables 1, 2).

**Figure 3:**
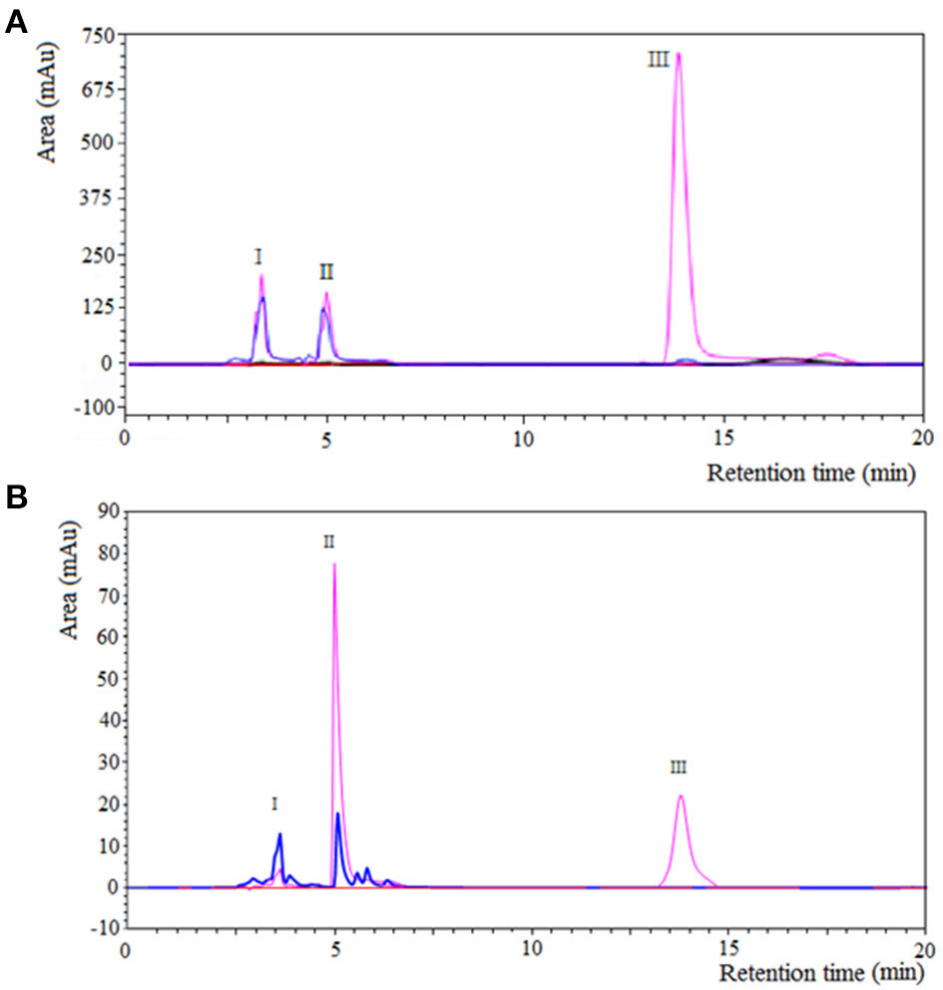
Component separation by RP-HPLC of a representative sample from Z-Tyr-Val-OH enzymatic synthesis under kinetic control, using: **(A)** antiacanthain (0.075 mg/ml, 1.875 IU/ml) and **(B)** granulosain (0.125 mg/ml, 1.875 IU/ml) as soluble biocatalysts, in 50% v/v ethyl ethanoate and 100 mM Tris(hydroxymethyl)amino methane hydrochloride buffer pH 8, after 4 h and 30 min of reaction, respectively; at 40°C under agitation at 200 rpm. I: antiacanthain (t_R_: 3–4.4 min) and granulosain (t_R_: 2,7–3,2 min); II: Z-Tyr-Val-OH (t_R_: 5.27 min); III: Z-Tyr-pNO (t_R_: 14.5 min). Aqueous phase: blue line. Organic phase: pink line.

**Figure 4:**
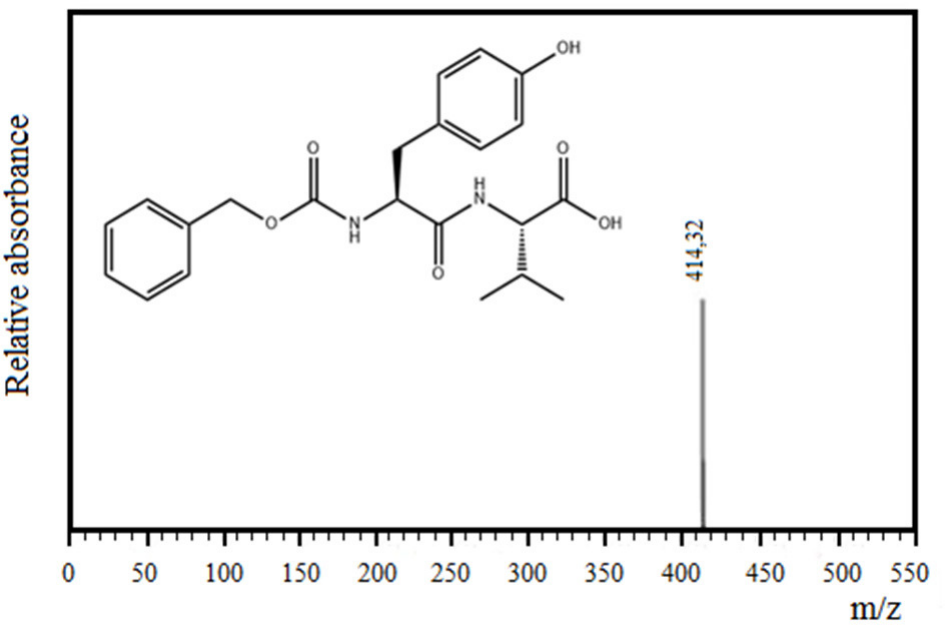
Mass spectrum of the carboxy terminal peptide Z-Tyr-Val-OH, m/z: 414.32.

Figures 3A,B shows the separation of components by RP-HPLC of two representative samples of the enzymatic synthesis of Z-Tyr-Val-OH, using antiacanthain and granulosain as soluble biocatalysts, after 4 h and 30 min of reaction, respectively. According to Figures 3A,B, at a retention time (t_R_) of 5.27 min, a peak of the main product (Z-Tyr-Val-OH) was observed in the organic phase. The product was easily separated from the organic layer after pausing the orbital shaker, and the synthetized peptide was purified (>95% purity) by chromatography, using a SPE-Cartridge C_18_. Figure 4 shows the mass spectrum of Z-Tyr-Val-OH, m/z: 414.32.

**Figure 5:**
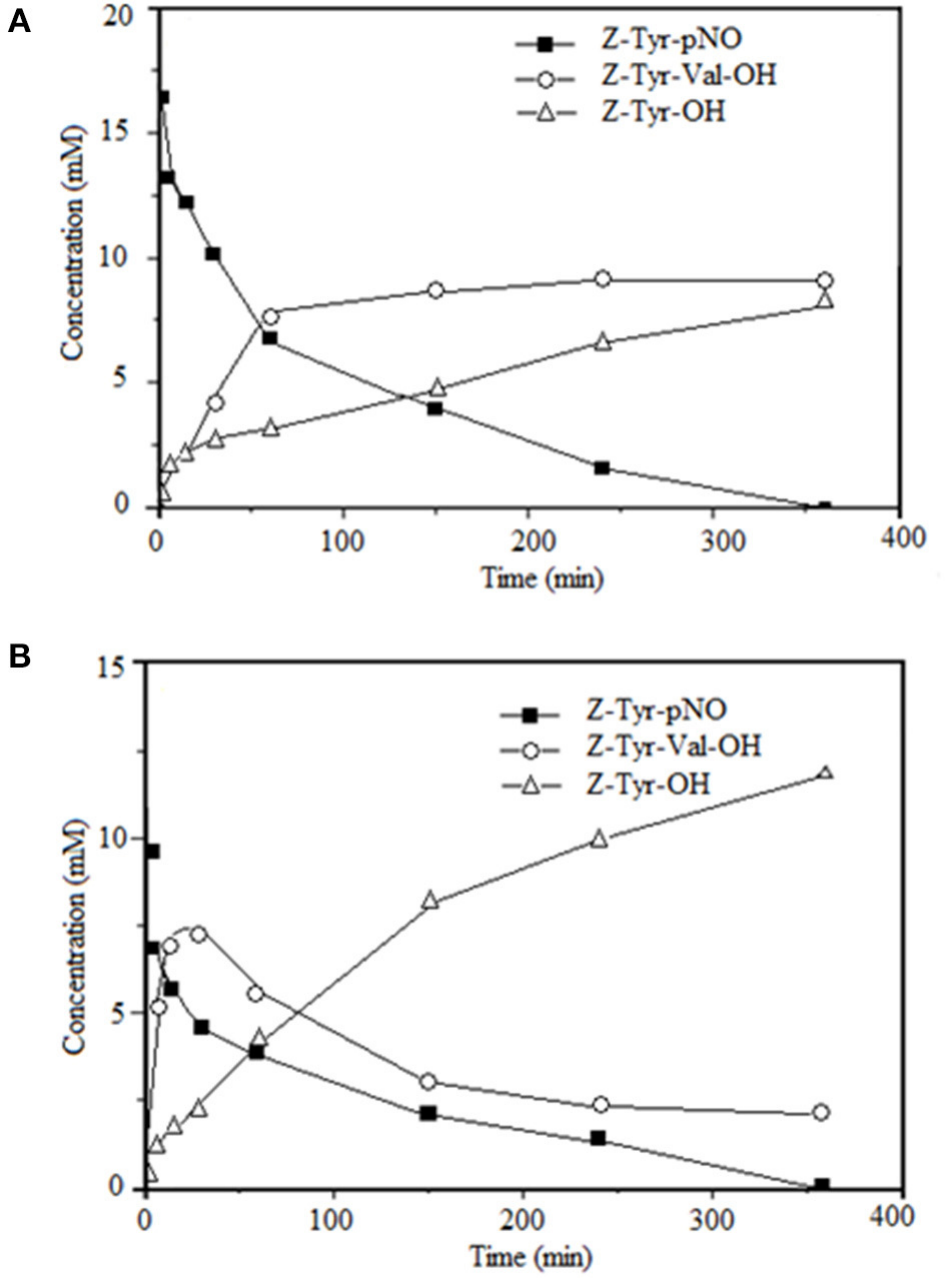
Time-course of the kinetically-controlled synthesis of Z-Tyr-Val-OH in 50% v/v ethyl ethanoate and 100 mM Tris(hydroxymethyl)amino methane hydrochloride buffer pH 8, at 40°C under agitation at 200 rpm; using: **(A)** antiacanthain (0.075 mg/ml, 1.875 IU/ml) and **(B)** granulosain (0.125 mg/ml, 1.850 IU/ml) as soluble biocatalysts.

Figures 5A,B shows the time-course of the reactions of synthesis of Z-Tyr-Val-OH, using antiacanthain and granulosain as soluble biocatalysts, respectively.

The only condensation product is converted into hydrolysis product (Z-Tyr-OH) after 30 min of reaction using granulosain as soluble biocatalysts. This effect was not observed when antiacanthain was used as biocatalyst and a maximum peptide concentration was obtained after 4 h without further hydrolysis. However, in both cases, the hydrolysis product (Z-Tyr-OH) was obtained from the acyl-donor substrate (Z-Tyr-pNO). It is well-known that, in the coupling reaction under kinetic control, the activated acyl donor becomes an intermediate, the so-called acyl-enzyme form, which reacts with the nucleophile for making the C-terminal segment of the peptide product. In a liquid-liquid biphasic system, the coupling reaction is carried out in the aqueous phase, where water can cleave the acyl-enzyme intermediate to give the hydrolyzed substrate (27). In fact, the hydrolysis of the acyl donor substrate decreased the product yields in the synthesis reaction of Z-Tyr-Val-OH, with either antiacanthain or granulosain as soluble biocatalysts.

Table 4 shows Z-Tyr-Val-OH product yields (η) of the enzymatic synthesis under kinetic control, using 50% v/v ethyl ethanoate in 100 mM Tris(hydroxymethyl)amino methane hydrochloride buffer pH 8 as reaction medium and soluble peptidases (antiacanthain and granulosain) as biocatalysts.

**Table 4:** Yields (η) obtained in the kinetically controlled synthesis of Z-Tyr-Val-OH, using 50% v/v ethyl ethanoate in 100 mM Tris(hydroxymethyl)amino methane hydrochloride buffer pH 8 as medium, and antiacanthain (0.075 mg/ml, 1.875 IU/ml) and granulosain (0.125 mg/ml, 1.850 IU/ml) as soluble biocatalysts, at 40°C under agitation at 200 rpm.

**Time (min)**	**Z-Tyr-Val-OH (mM)**		**α_s_ (%)**		*****η***** **(%)**	
	**Antiacanthain Granulosain**		**Antiacanthain Granulosain**		**Antiacanthain Granulosain**	
0	0^b^	0^b^	0^a^	0^a^	0^a^	0^a^
1	0.35 ± 0.03^c^	4.16 ± 0.05^e^	4.6 ± 0.09^b^	31.4 ± 0.05^b^	2.45 ± 0.09^b^	29.7 ± 0.03^f^
5	1.75 ± 0.20^d^	5.22 ± 0.10^f^	23.2 ± 0.33^c^	51.4 ± 0.10^c^	10.1 ± 0.10^c^	37.3 ± 0.07^g^
15	2.20 ± 0.01^e^	6.80 ± 0.05^g^	29.4 ± 0.19^d^	60.0 ± 0.25^d^	12.75 ± 0.05^d^	48.6 ± 0.03^h^
30	4.30 ± 0.01^f^	7.30 ± 0.10^h^	41.3 ± 0.12^e^	67.6 ± 0.09^e^	24.93 ± 0.12^e^	**52.1** **±** **0.10**^i^
60	7.53 ± 0.10^g^	3.50 ± 0.09^d^	63.1 ± 0.20^f^	80.6 ± 0.45^f^	43.65 ± 0.09^f^	25.0 ± 0.15^e^
150	8.53 ± 0.05^h^	3.00 ± 0.10^c^	76.9 ± 0.07^g^	84.6 ± 0.52^g^	49.45 ± 0.05^g^	21.4 ± 0.09^d^
240	9.22 ± 0.01^a^	2.35 ± 0.01^a^	91.3 ± 0.05^h^	88.1 ± 0.09^h^	**53.45** **±** **0.01**^h^	16.8 ± 0.05^c^
360	9.02 ± 0.12^a^	2.10 ± 0.20^a^	100 ± 0.01^i^	100 ± 0.01^i^	52.28 ± 0.05^i^	15.0 ± 0.10^b^

*Acyl acceptor/donor molar ratio: 1. α_s_ is the conversion percentage of acyl donor substrate*.

*The values with different superscript letters in a column are significantly different (p < 0.05) (IBM SPSS Statistics 27.0)*.

*Bold values represent the maximal peptide yields from each experimental trial set*.

Soluble antiacanthain showed a slightly higher peptide yield than soluble granulosain, under those conditions. However, soluble granulosain showed higher peptide productivity (14.6 ± 0.01 mM/h) than soluble antiacanthain (2.30 ± 0.015 mM/h), but 58% of the substrate was rapidly hydrolyzed to Z-Tyr-OH preventing the synthesis could continue.

It is noteworthy that equimolecular substrate concentrations in the aqueous phase of the liquid-liquid biphasic media were used in those trials. Consequently, two strategies were applied to maximize η: (1) the acyl acceptor concentration was increased until its maximum solubility (75.5 mM); and (2) Immobilized enzymes (antiacanthain and granulosain) by multi-point covalent bonding in glyoxyl-silica were applied as biocatalysts of the peptide synthesis reaction in both selected media: 50% v/v ethyl ethanoate in 100 mM Tris(hydroxymethyl)amino methane hydrochloride buffer pH 8 and almost anhydrous ethyl ethanoate (X_w_: 1 × 10^−5^).

Under the first strategy, at acyl acceptor/donor molar ratio of 10, the peptide yields were 72 ± 0.15 and 60 ± 0.10% using soluble antiacanthain and granulosain, respectively (Table 5). These values were 35 and 15% higher than those obtained under equimolar concentrations of both reactants, using 50% v/v ethyl ethanoate in 100 mM Tris hydrochloride buffer pH 8.0 as reaction medium (Table 4). The maximum peptide productivity (20.7 ± 0.015 mM/h) was obtained using soluble granulosain as biocatalyst in the same reaction medium.

**Table 5:** Yields (η) obtained in the kinetically controlled synthesis of Z-Tyr-Val-OH, using 50% v/v ethyl ethanoate in 100 mM Tris(hydroxymethyl)amino methane hydrochloride buffer pH 8 as medium, and antiacanthain (0.075 mg/ml, 1.875 IU/ml) and granulosain (0.125 mg/ml, 1.850 IU/ml) as soluble biocatalysts, at 40°C under agitation at 200 rpm.

**Time (min)**	**Z-Tyr-Val-OH (mm)**		**α_s_ (%)**		*****η***** **(%)**	
	**Antiacanthain Granulosain**		**Antiacanthain Granulosain**		**Antiacanthain Granulosain**	
0	0^b^	0^a^	0^a^	0^b^	0^a^	0^a^
1	0.57 ± 0.32^c^	2.45 ± 0.02^b^	3.77 ± 0.10^b^	14.2 ± 0.05^c^	3.29 ± 0.0^b^	14.2 ± 0.00^b^
5	1.83 ± 0.01^d^	6.16 ± 0.23^f^	10.6 ± 0.32^c^	35.7 ± 0.01^d^	10.6 ± 0.05^c^	35.7 ± 0.01^f^
15	4.23 ± 0.05^e^	8.16 ± 0.03^h^	24.5 ± 0.01^d^	52.7 ± 0.35^e^	24.5 ± 0.10^d^	47.3 ± 0.05^h^
30	6.21 ± 0.05^f^	10.3 ± 0.05^i^	36.0 ± 0.14^e^	76.3 ± 0.05^f^	36.0 ± 0.02^e^	**60.0** **±** **0.05**^i^
60	10.2 ± 0.01^g^	7.33 ± 0.05^g^	73.3 ± 0.05^f^	81.2 ± 0.51^g^	59.3 ± 0.01^f^	42.5 ± 0.03^g^
150	11.9 ± 0.07^h^	5.71 ± 0.21^e^	89.6 ± 0.45^g^	93.5 ± 0.03^h^	68.7 ± 0.05^g^	33.1 ± 0.20^e^
240	12.4 ± 0.01^a^	4.23 ± 0.05^d^	98.0 ± 0.05^h^	100 ± 0.00^a^	**72.0** **±** **0.01**^h^	24.5 ± 0.05^d^
360	12.2 ± 0.25^a^	3.67 ± 0.05^c^	100 ± 0.00^i^	100 ± 0.00^a^	70.8 ± 0.05^i^	21.0 ± 0.20^c^

*Acyl acceptor/donor molar ratio: 10. α_s_ is the conversion percentage of acyl donor substrate*.

*The values with different superscript letters in a column are significantly different (p < 0.05) (IBM SPSS Statistics 27.0)*.

*Bold values represent the maximal peptide yields from each experimental trial set*.

The synthesis of Z-Tyr-Val-OH did not work in almost anhydrous ethyl ethanoate (X_w_: 1 × 10^−5^), under the same reaction conditions, because the soluble peptidases (antiacanthain and granulosain) were quickly inactivated.

Under the second strategy, the multi-point immobilization of antiacanthain and granulosain in glyoxyl-silica did not lead to better peptide yields than soluble enzymes, using the liquid-liquid biphasic medium formed by 50% v/v ethyl ethanoate in 100 mM Tris(hydroxymethyl)amino methane hydrochloride buffer pH 8. However, immobilized antiacanthain prove to be a robust biocatalyst in almost anhydrous ethyl ethanoate (X_w_: 1 × 10^−5^), eliciting a maximum peptide yield of 75 ± 0.13% (Table 6). Immobilized granulosain was inactivated in almost anhydrous ethyl ethanoate, under the same reaction conditions.

**Table 6:** Yields (η) obtained in the kinetically controlled synthesis of Z-Tyr-Val-OH in ethyl ethanoate (X_w_: 1 × 10^−5^ in the enzyme into the organic solvent) at 40°C, using glyoxyl-silica immobilized antiacanthain (12.5 mg/ml) as biocatalyst.

**Time (min)**	**Z- Tyr-pNO (mM)**	**Z-Tyr-Val-OH (mM)**	**α_s_ (%)**	**η (%)**
0	17.25	0	0^a^	0^a^
5	14.83	2.41	14 ± 0.20^b^	14 ± 0.00^b^
15	12.94	4.30	25 ± 0.37^c^	25 ± 0.01^c^
30	10.35	6.90	40 ± 0.00^d^	40 ± 0.15^d^
60	9.15	8.20	47 ± 0.10^e^	47± 0.03^e^
240	4.80	12.10	72 ± 0.00^f^	70 ± 0.02^f^
360	4.25	12.94	75 ± 0.18^g^	**75** **±** **0.13**^g^

*α_s_ is the conversion percentage of acyl donor substrate*.

*The values with different superscript letters in a column are significantly different (p < 0.05) (IBM SPSS Statistics 27.0)*.

*Bold values represent the maximal peptide yields from each experimental trial set*.

Table 6 shows that the acyl-donor ester was neither completely consumed nor hydrolyzed after 6 h. This fact was due to the faster deactivation of antiacanthain-glyoxyl-silica in the almost anhydrous organic solvent than in the liquid-liquid biphasic system. The direct effect of the organic solvent on the enzyme could be the main reason of deactivation. The literature reports that other enzymes in almost anhydrous organic solvents showed an increased structural rigidity which led to a lower reactivity (27, 34). The substrate solvation (into the microenvironment of the enzyme active site) does not seem to be the deactivation cause because as the reaction progresses the amount of water produced also increases. Besides, internal mass transfer limitations of the substrate (due to a higher reaction rate than the substrate diffusion rate) have not been observed when glyoxyl silica immobilized antiacanthain was placed in almost anhydrous ethyl ethanoate (X_w_: 1 × 10^−5^). In effect, the peptide productivity in this system was 2.16 ± 0.01 mM/h, a value close to that obtained in 50% v/v ethyl ethanoate in 100 mM Tris(hydroxymethyl)amino methane hydrochloride buffer pH 8.

Finally, under thermodynamic control, both soluble and immobilized peptidases (antiacanthain and granulosain) did not catalyze the synthesis of Z-Tyr-Val-OH peptide in 50% v/v ethyl ethanoate in 100 mM Tris(hydroxymethyl)amino methane hydrochloride buffer pH 8 and in almost anhydrous ethyl ethanoate, despite of the high selectivity showed by both enzymes under kinetic control in those media, where no by-products were formed. This fact showed that these enzymes do not work against not activated acyl donors, such as Z-Tyr-OH.

### Antibacterial Activity of Z-Tyr-Val-OH

**Figure 6:**
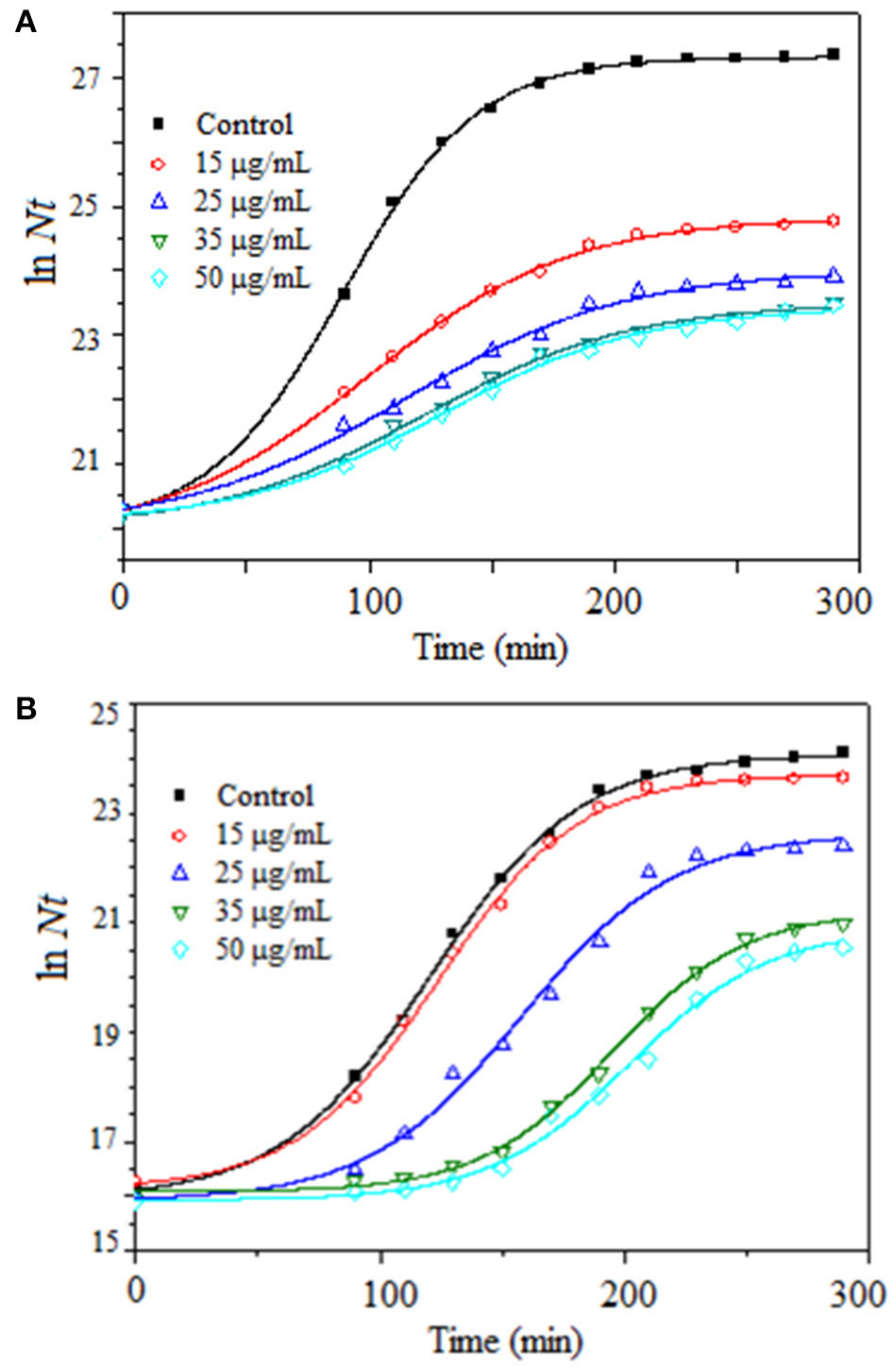
Growth kinetics of **(A)** *Staphylococcus aureus* ATCC 25923 and **(B)** *Escherichia coli* ATCC 25922 in batch cultures, at 37°C under agitation at 180 rpm, using Müller-Hinton broth and several Z-Tyr-Val-OH concentrations within the range from 0 to 50 μg/ml.

Antibacterial activity of Z-Tyr-Val-OH was tested through three independent trials, in duplicate, against *S. aureus* ATCC 25923 and *E. coli* ATCC 25922 as reference strains in batch culture with Müller-Hinton broth at 37°C under agitation at 180 rpm, using increasing concentrations of Z-Tyr-Val-OH (0–50 μg/ml) (Figure 6).

According to the Kruskal-Wallis method, the Z-Tyr-Val-OH peptide at all concentrations tested produced a significant decrease (*p* ≤ 0.05) in specific growth rates (mean values) of *S. aureus* with respect to the control (Table 7). Besides, Z-Tyr-Val-OH showed a significant decrease (*p* ≤ 0.05) in the specific growth rates of *E. coli*, with respect to the control, at ≥ 35 μg/ml.

**Table 7:** Statistical analysis by the Kruskal – Wallis test of the specific growth rate in batch culture of *Staphylococcus aureus* ATCC 25923 and *Escherichia coli* ATCC 25922, using Müller-Hinton broth and the addition of different concentrations of Z-Tyr-Val-OH, at 37°C under agitation at 180 rpm.

**Z-Tyr-Val-OH (μg/ml)**	**Kruskal-Wallis test (*p*)**	**Specific growth rate (h^**−1**^)**
***Staphylococcus aureus*** **ATCC 25923**		
0	–	4.26 ± 0.11
15	0.050	1.62 ± 0.15
25	0.001	1.25 ± 0.20
35	0.000	1.02 ± 0.18
50	0.000	1.00 ± 0.17
***Escherichia coli*** **ATCC 25922**		
0	–	3.68 ± 0.17
15	0.100	3.15 ± 0.19
25	0.600	2.47 ± 0.15
35	0.023	1.74 ± 0.20
50	0.004	1.14 ± 0.015

Table 8 shows the minimum inhibitory concentrations (MICs) values of Z-Tyr-Val-OH against the reference strains (*S. aureus* and *E. coli*) and six wild strains isolated in the Central Microbiology Laboratory of the San Luis Ministry of Health, Government of San Luis, Province of San Luis, Argentina.

**Table 8:** MICs of Z-Tyr-Val-OH peptide determined for Gram positive and Gram negative strains.

**Microorganism**	MICs^*^		
	**Z-Tyr-Val-OH (μg/ml)**	**Oxacillin (μg/ml)**	**Nalidixic acid (μg/ml)**
**Gram positive bacteria**			
*Staphylococcus aureus* ATCC 25923	47.5 ± 0.01^b^	31.1 (47)	
*Staphylococcus aureus* C00195	96.0 ± 0.00^a^		
*Staphylococcus hominis* A17771	96.0 ± 0.05^a^		
*Enterococcus faecalis* I00125 (MDR)	—		
**Gram negative bacteria**			
*Escherichia coli* ATCC 25922	55.0 ± 0.01^a^		21.7 (48)
*Escherichia coli* A17683	123.0 ± 0.10^b^		
Klebsiella oxytoca A19438 (SDR)	140.0 ± 0.05^c^		
*Pseudomonas aeruginosa* C00213 (SDR)	—		

* *MICs data are representative of three independent experiments which were done in duplicate. MDR, multidrug resistance; SDR, single-drug resistance. The values with different superscript letters in a column are significantly different (p < 0.05) (IBM SPSS Statistics 27.0)*.

MICs of Z-Tyr-Val-OH against the reference strains, *S. aureus* and *E. coli*, were 47.5 ± 0.01 and 55 ± 0.01 μg/ml, respectively. Those MICs values were higher than those reported by oxacillin (31.1 μg/ml) or nalidixic acid (21.7 μg/ml) under similar culture conditions (46, 47).

All Gram positive and Gram negative wild strains sensitive to commonly used antibiotics, such as *Staphylococcus aureus* C00195, *Staphylococcus hominis* A17771 and *E. coli* A17683, were also susceptible to Z-Tyr-Val-OH. MICs values of these wild strains sensitive to commonly used antibiotics ranged from 96.0 ± 0.00 to 123.0 ± 0.10 μg/ml. Besides, *Klebsiella oxytoca* A19438, a Gram negative wild strain with natural or intrinsic resistance to ampicillin, was also susceptible to Z-Tyr-Val-OH at ≥ 140.0 ± 0.05 μg/ml.

Conversely, Z-Tyr-Val-OH was not effective within the ranges tested against bacteria with single resistance to carbapenems and with multiple resistances to vancomycin and β-lactam antibiotics, such as *Pseudomonas aeruginosa* C00213 and *Enterococcus faecalis* I00125, respectively.

It is essential to highlight that the Z-Tyr-Val-OH sequence has not yet been reported in bioactive peptide databases, such as: PepBank (58), PeptideDB (59), BIOPEP (60); nor in the databases of antimicrobial peptides, such as: APD3, Antimicrobial Peptide Database (61), and CAMP (62).

## Conclusions

This work reports two partially purified proteolytic extracts from the fruits of *Bromelia antiacantha* and *Solanum granulosum leprosum*, belonging to the papain-like family of peptidases, as new biocatalysts for the green synthesis of bioactive peptides.

Despite their similar nature, antiacanthain showed different behavior than granulosain for the production of the novel antibacterial dipeptide Z-Tyr-Val-OH. Soluble antiacanthain catalyzed the dipeptide synthesis under kinetic-control with high yield (72%) in 50% v/v ethyl ethanoate in 100 mM Tris(hydroxymethyl)amino methane hydrochloride buffer pH 8, while glyoxyl-silica immobilized antiacanthain allow to obtain the best peptide yield (75%) in almost anhydrous ethyl ethanoate (X_w_: 1 × 10^−5^). Soluble granulosain showed slightly lower peptide yields and higher peptide productivities than soluble antiacanthain in that liquid-liquid biphasic medium, but soluble and immobilized granulosain were inactivated in the almost anhydrous ethyl ethanoate (X_w_: 1 × 10^−5^). Both enzymes were highly selective for the synthesis of Z-Tyr-Val-OH, and no other products were synthesized.

Glyoxyl-silica immobilized antiacanthain showed a much better performance than the soluble enzyme in almost anhydrous organic solvents. However, other immobilization methodologies need to be investigated to decrease the enzyme's inactivation rates under those hard reaction conditions, for leading to more sustainable production processes.

Nowadays, the molecular effect of organic solvents on the activity and stability of antiacanthain and granulosain, and changes related to solvation, flexibility and secondary structure of those enzymes in different aqueous-organic biphasic media and in almost anhydrous organic solvents are being evaluated.

This work reports a novel broad-spectrum antibacterial peptide (Z-Tyr-Val-OH) that significantly decreased (*p* ≤ 0.05) the specific growth rates of Gram positive and Gram negative reference bacteria at very low concentrations of ≥15 and 35 μg/ml, respectively.

Z-Tyr-Val-OH also showed high efficacy at low concentrations against Gram positive and Gram negative wild strains sensitive to conventional antibiotics. Furthermore, the novel dipeptide was very effective at ≥140.0 ± 0.05 μg/ml against Gram negative wild bacteria with single resistance to ampicillin, such as *K. oxytoca*. This last antimicrobial activity of Z-Tyr-Val-OH gives relevance and novelty to this work because *K. oxytoca* is an emerging multi-drug resistant bacterium in the hospital-acquired infections by adults.

Conversely, the dipeptide did not demonstrated efficacy against bacteria with single resistance to carbapenems and with multiple resistances to vancomycin and β-lactam antibiotics, within the range tested.

Finally, this study contributes with a new efficient and safe food preservative to be applied to different real food systems.

## Data Availability Statement

The original contributions presented in the study are included in the article, further inquiries can be directed to the corresponding author.

## Author Contributions

SB designed the experiments, did the data analyzing, and manuscript writing. MA and GB did the experimental assays, data collection, and analysis. JT and FG collaborated with MA and GB in the antimicrobial activity experiments and analysis of peptides, respectively. DV and CB collaborated with MA and GB in the preparation of partially purified enzymatic extracts and enzyme immobilization methodology, respectively. All authors contributed to the article and approved the submitted version.

## Conflict of Interest

The authors declare that the research was conducted in the absence of any commercial or financial relationships that could be construed as a potential conflict of interest.

## References

[1] Kosikowska P Lesner A. Antimicrobial peptides (AMPs) as drug candidates: a patent review (2003-2015). Expert Opin Ther Pat. (2016) 26:689–702. 10.1080/13543776.2016.1176149 27063450

[2] Barberis S Quiroga HG Barcia C Talia JM Debattista N. Natural food preservative against microorganisms. In: Grumezescu AM Holban AM. Food Safety and Preservation: Modern Biological Approaches to Improving Consumer Health. London: Elsevier Inc. (2018). p. 621–9. 10.1016/B978-0-12-814956-0.00020-2

[3] Deep A Chaudhary U Gupta V. Quorum sensing and bacterial pathogenicity: from molecules to disease. J Lab Physicians. (2011) 3:4–11. 10.4103/0974-2727.78553 21701655 PMC3118056

[4] Weiss J Zhong Q Harte F Davidson M. Micro- and nanoparticles for controlling microorganisms in foods. In: Pabst G Kučerka N Nieh M Katsaras J. Liposomes, Lipid Bilayers and Model Membranes: from Basic Research to Technology. Florida: CRC Press (2016). p. 416–25.

[5] Pérez Pulido R Grande Burgos MJ Gálvez A Lucas López R. Application of bacteriophages in post-harvest control of human pathogenic and food spoiling bacteria. Crit Rev Biotechnol. (2016) 35:851–61. 10.3109/07388551.2015.1049935 26042353

[6] Torres-Barceló C. The disparate effects of bacteriophages on antibiotic-resistant bacteria. Review. Emerg Microbes Infect. (2018) 7:168. 10.1038/s41426-018-0169-z 30302018 PMC6177407

[7] Barberis S Origone A Adaro M Bersi G. Bioactive peptides as functional food ingredients. In: Kastin A. Handjournal of Food Bioengineering. London: Elsevier Inc. (2018). p. 147–74. 10.1016/B978-0-12-811448-3.00005-X

[8] World Health Organization. JECFA. (2010). Available online at: http://apps.who.int/food-additives-contaminants-jecfa-database/ (accessed December 8, 2020).

[9] Olatunde OO Benjakul S. Natural preservatives for extending the shelf-life of seafood: a Revisit. Compr Rev Food Sci Food Saf. (2018) 17:1595–12. 10.1111/1541-4337.12390 33350137

[10] Ahmed TAE Hammami R. Recent insight into structure-function relationships of antimicrobial peptide. Review. J Food Biochem. (2019) 43:e12546. 10.1111/jfbc.12546 31353490

[11] Barberis S Adaro M Origone A Bersi G Guzmán F Illanes A. Peptide synthesis using proteases as catalyst. In: Guevara MG Daleo GR. Biotechnological Applications of Plant Proteolytic Enzymes. Basel: Springer Nature (2018). p. 69–106. 10.1007/978-3-319-97132-2_4

[12] Zhang LJ Gallo RL. Antimicrobial peptides. Curr Biol. (2016) 26:R1–21. 10.1016/j.cub.2015.11.017 26766224

[13] Fjell CD Hiss JA Hancock REW Schneider G. Designing antimicrobial peptides: form follows function. Nat Rev Drug Discov. (2012) 11:37–51. 10.1038/nrd3591 22173434

[14] Marcos JF Muñoz A Pérez-Payá A Misra S López-García B. Identification and rational design of novel antimicrobial peptides for plant protection. Annu Rev Phytopathol. (2008) 46:273–301. 10.1146/annurev.phyto.121307.094843 18439131

[15] Lee Ventola C. The antibiotic resistance crisis. Pharm Ther. (2015) 40:277–83. PMC4378521 25859123

[16] Friedrich CL Moyles D Beveridge TJ Hancock REW. Antibacterial action of structurally diverse cationic peptides on Gram-positive bacteria. Antimicrob Agents Chemother. (2000) 44:2086–92. 10.1128/AAC.44.8.2086-2092.2000 10898680 PMC90018

[17] Álvarez CA Guzmán F Cárdenas C Marshall SH Mercado L. Antimicrobial activity of trout hepcidin. Fish Shellfish Immun. (2014) 41:93–101. 10.1016/j.fsi.2014.04.013 24794583

[18] Jenssen H. Anti-herpes simplex virus activity of lactoferrin/lactoferricin an example of antiviral activity of antimicrobial protein/peptide. Cell Mol Life Sci. (2005) 62:3002–13. 10.1007/s00018-005-5228-7 16261265 PMC11139097

[19] Marr AK Gooderham WJ Hancock REW. Antibacterial peptides for therapeutic use: obstacles and realistic outlook. Curr Opin Pharmacol. (2006) 6:468–72. 10.1016/j.coph.2006.04.006 16890021

[20] Ortiz-Martínez M Winkler R García-Lara S. Preventive and therapeutic potential of peptides from cereals against cancer. J Proteomics. (2014) 111:165–83. 10.1016/j.jprot.2014.03.044 24727098

[21] Agyei D Danquah K. Industrial-scale manufacturing of pharmaceutical-grade bioactive peptides. Biotechnol Adv. (2012) 29:272–77. 10.1016/j.biotechadv.2011.01.001 21238564

[22] Wall RJ Kerr DE Bondioli KR. Transgenic dairy cattle: genetic engineering on a large scale. J Dairy Sci. (1997) 80:2213–24. 10.3168/jds.S0022-0302(97)76170-8 9313167

[23] Wakasa Y Tamakoshi C Ohno T Hirose S Goto T Nagaoka S. The hypocholesterolemic activity of transgenic rice seed accumulating lactostatin, a bioactive peptide derived from bovine milk β-lactoglobulin. J Agr Food Chem. (2011) 59:3845–50. 10.1021/jf200044j 21410288

[24] Hartsough EM Shah P Larsen AC Chaput JC. Comparative analysis of eukaryotic cell - free expression systems. BioTechniques. (2015) 59:149–51. 10.2144/000114327 26345507

[25] Limón R Peñas E Torino M Martínez-Villaluenga C Dueñas M Frias J. Fermentation enhances the content of bioactive compounds in kidney bean extracts. Food Chem. (2015) 172:343–52. 10.1016/j.foodchem.2014.09.084 25442563

[26] Fosgerau K Hoffmann T. Peptide therapeutics: current status and future directions. Review. Drug Discov Today. (2015) 20:122–8. 10.003 10.1016/j.drudis.2014.10.003 25450771

[27] Barberis S Guzmán F Illanes A López-Santín J. Enzyme biocatalysis: principles and applications. In: Illanes A. Study Cases of Enzymatic Processes. Berlin: Springer Science + Business Media B.V. (2008). p. 253–73. 10.1007/978-1-4020-8361-7_6

[28] Adaro MO Vallés D Cantera AM Talia JM Barberis S. Antibacterial activity of the proteolytic extract from fruits of *Solanum granuloso-leprosum* (Solanaceae). Lat Am J Pharm. (2019) 38:2032–35.

[29] Bersi G Valles D Penna F Cantera AM Barberis S. Valorization of fruit by-products of *Bromelia antiacantha* Bertol.: protease obtaining and its potential as additive for laundry detergents. Biocatal Agric Biotechnol. (2019) 18:101099. 10.1016/j.bcab.2019.101099

[30] Vallés D Cantera AM. Antiacanthain a: new proteases isolated from *Bromelia antiacantha* Bertol. (Bromeliaceae). Int J Biol Macromol. (2018) 113:916–23. 10.1016/j.ijbiomac.2018.03.025 29522824

[31] Vallés D Furtado S Cantera AMB. Characterization of news proteolytic enzymes from ripe fruits of *Bromelia antiacantha* Bertol. (Bromeliaceae). Enzyme Microb Technol. (2007) 40:409–13. 10.1016/j.enzmictec.2006.07.011

[32] Vallés D Bruno M López LMI Caffini N Cantera AM. Granulosain I, a cystein protease isolated from ripe fruits of *Solanum granuloso-leprosum* (Solanaceae). Protein J. (2008) 27:267–75. 10.1007/s10930-008-9133-4 18478320

[33] Vallés D Furtado S Villadonica C Cantera AMB. Characterisation, stabilisation and possible biotechnological applications of new proteolytic enzymes from *Solanum granuloso-leprosum*. Int J Biotechnol. (2004) 6:346–60. 10.1504/IJBT.2004.005517

[34] Illanes A Guzmán F Barberis S. Proteases as powerful catalysts for organic synthesis. In: Hughes AB. Part Three: Enzymes. Amino Acids, Peptides and Proteins in Organic Chemistry. Weinheim: Wiley-VCH (2009). p. 341–77. 10.1002/9783527631780.ch8

[35] Barbosa O Ortiz C Berenguer-Murcia Á Torres R Rodrigues RC Fernandez-Lafuente R. Strategies for the one-step immobilization-purification of enzymes as industrial biocatalysts. Review. Biotechnol Adv. (2015) 33:435–56. 10.1016/j.biotechadv.2015.03.006 25777494

[36] Boudrant J Woodley JM Fernández-Lafuente R. Parameters necessary to define an immobilized enzyme preparation. Process Biochem. (2020) 90:66–80. 10.1016/j.procbio.2019.11.026

[37] Mateo C Abian O Fernandez-Lorente G Pessela BCC Grazu V Guisan JM. Multi-point covalent immobilization of enzymes on supports activated with epoxy groups: stabilization of industrial enzymes. In: Guisan J Bolivar J López-Gallego F Rocha-Martín J. Immobilization of Enzymes and Cells. Methods in Molecular Biology. New York, NY: Humana (2020). vol. 2100; p. 109–. 10.1007/978-1-0716-0215-7_6 31939118

[38] Schagger H Von Jagow G. Tricine-sodium dodecyl sulfate-polyacrylamide gel electrophoresis for the separation of proteins in the range from 1 to 100 kDa. Anal Biochem. (1987) 166:368–79. 10.1016/0003-2697(87)90587-2 2449095

[39] Bradford MM. A rapid and sensitive method for the quantitation of microgram quantities of protein utilizing the principles of protein-dye binding. Anal Biochem. (1976) 7:248–54. 10.1016/0003-2697(76)90527-3 942051

[40] Barberis S Quiroga E Morcelle S Priolo N Luco JM. Study of phytoproteases stability in aqueous-organic biphasic systems using linear free energy relationships. J Mol Catal B Enzym. (2006) 38:95–103. 10.1016/j.molcatb.2005.11.011

[41] Urrutia P Mateo C Guisan JM Wilson L Illanes A. Immobilization of *Bacillus circulans* β-galactosidase and its application in the synthesis of galacto-oligosaccharides under repeated-batch operation. Biochem Eng J. (2013) 77:41–8. 10.1016/j.bej.2013.04.015

[42] Zaks A Klibanov AM. The effect of water on enzyme action in organic media. J Biol Chem. (1988) 263:8017–21. 10.1016/S0021-9258(18)68435-2 3131337

[43] Priolo N Arribére MC Caffini N Barberis S Nieto Vázquez R Luco JM. Isolation and purification of cysteine peptidases from the latex of *Araujia hortorum* fruits. Study of their esterase activities using partial least-squares (PLS) modeling. J Mol Catal B-Enzym. (2001) 15:177–89. 10.1016/S1381-1177(01)00022-4

[44] Bernal C Sierra L Mesa M. Improvement of thermal stability of β-galactosidase from *Bacillus circulans* by multipoint covalent immobilization in hierarchical macro-mesoporous silica. J Mol Catal B Enzym. (2012) 84:166–72. 10.1016/j.molcatb.2012.05.023

[45] Origone A Bersi G Illanes A Sturniolo H Liggieri C Guzmán F. Enzymatic and chemical synthesis of new anticoagulant peptides. Biotechnol Prog. (2018) 34:1093–101. 10.1002/btpr.2658 29882241

[46] Talia JM Debattista NB Pappano N. Susceptibility of *Staphylococcus aureus* strains toward combinations of oxacillin-2,4-dihydroxychalcone. Folia Microbiol. (2009) 54:516–20. 10.1007/s12223-009-0074-x 20140719

[47] Talia JM Debattista N Pappano N. New antimicrobial combinations: substituted chalcones-oxacillin against methicillin resistant *Staphylococcus aureus*. Braz J Microbiol. (2011) 42:470–5. 10.1590/S1517-83822011000200010 24031657 PMC3769842

[48] Talia JM Tonn CE Debattista NB Pappano NB. Antibacterial efficacy of dihydroxylated chalcones in binary and ternary combinations with nalidixic acid and nalidix acid–rutin against *Escherichia coli* ATCC 25922. Indian J Microbiol. (2012) 52:638–41. 10.1007/s12088-012-0302-y 24293723 PMC3516650

[49] McDonald JH. Handjournal of Biological Statistics in: Kruskall-Wallis Test. Baltimore: Sparky House Publishing (2014). p. 157–64.

[50] Santos VNC De Freitas RA Deschamps FC Biavatti MW. Ripe fruits of *Bromelia antiacantha*: investigations on the chemical and bioactivity profile. Braz J Pharmacogn. (2009) 19:358–65. 10.1590/S0102-695X2009000300004

[51] Levate Macedo L da Silva Araújo C Ribeiro Vilela D Fonseca HC Vilela Goulart NM de Barros Vilas Boas EV. Effect of maturation stage on the physical-chemical composition and bioactive compounds of *Solanum granosos-leprosum* Dunal fruits. Res Soc Dev. (2020) 9:e22996323. 10.33448/rsd-v9i9.6323

[52] Bernal C Illanes A Wilson L. Heterofunctional hydrophilic-hydrophobic porous silica as support for multipoint covalent immobilization of lipases: application to lactulose palmitate synthesis. Langmuir. (2014) 12:3557–66. 10.1021/la4047512 24621332

[53] Quiroga E Priolo N Marchese J Barberis S. Stability of *araujiain*, a novel plant protease, in different organic systems. Acta Farm Bonaerense. (2005) 24:204–8. Available online at: http://www.latamjpharm.org/resumenes/24/2/LAJOP_24_2_1_6.pdf

[54] Illanes A Cauerhff A Wilson L Castro GR. Recent trends in biocatalysis engineering. Bioresour Technol. (2013) 115:48–57. 10.1016/j.biortech.2011.12.050 22424920

[55] Adaro MO. Enzymatic synthesis of antimicrobial peptides useful as new food preservatives (doctoral thesis). National University of San Luis. San Luis, Argentina (2020).

[56] Yang L Dordick JS Garde S. Hydration of enzymes in non-aqueous media is consistent with solvent dependence of its activity. Biophys J. (2004) 87:812–21. 10.1529/biophysj.104.041269 15298890 PMC1304491

[57] Schechter I Berger A. On the size of the active site in proteases. I. Papain. Biochem Biophys Res Commun. (1967) 27:157–62. 10.1016/S0006-291X(67)80055-X 6035483

[58] Shtatland T Guettler D Kossodo M Pivovarov M Weissleder R. PepBank a database of peptides based on sequence text mining and public peptide data sources. BMC Bioinformatics. (2007) 8:280. 10.1186/1471-2105-8-280 17678535 PMC1976427

[59] Liu F Baggerman G Schoofs L Wets G. The construction of a bioactive peptide database in metazoan. J Proteome Res. (2008) 7:4119–31. 10.1021/pr800037n 18707150

[60] Dziuba J Minkiewicz P Nalecz D Iwaniak A. Database of biologically active peptide sequence. Nahrung. (1999) 43:190–95. 10.1002/(SICI)1521-3803(19990601)43:3 10399353

[61] Wang G Li X Wang XLZ. APD3: the antimicrobial peptide database as a tool for research and education. Nucleic Acids Res. (2017) 44:1087–93. 10.1093/nar/gkv1278 26602694 PMC4702905

[62] Thomas S Karnik S Shankar Barai R Jayaraman VK Idicula-Thomas S. CAMP: a useful resource for research on antimicrobial peptides. Nucleic Acids Res. (2010) 38:774–80. 10.1093/nar/gkp1021 19923233 PMC2808926

